# Effect of Perfluorooctanesulfonic Acid on Fibrin Clot Properties and Thrombin Generation: Linking Environmental Pollution with Cardiovascular Diseases

**DOI:** 10.3390/jcdd13050209

**Published:** 2026-05-13

**Authors:** Jakub Kuciński, Krzysztof Krawczyk, Konrad Kieca, Aleksander Siniarski, Michał Ząbczyk, Ewa Konduracka

**Affiliations:** 1 Department of Coronary Disease and Heart Failure, The St. John Paul II Hospital, 31-202 Krakow, Poland; lek.kucinski@gmail.com (J.K.); krzysztof15.krawczyk@gmail.com (K.K.); aleksandersiniarski@gmail.com (A.S.); 2 Faculty of Medicine and Health Sciences, Medical College, Jagiellonian University, 30-060 Krakow, Poland; michalzabczyk@op.pl; 3 Faculty of Chemistry, Jagiellonian University, 30-060 Krakow, Poland; konrad.kieca@uj.edu.pl; 4 Doctoral School of Exact and Natural Sciences, Jagiellonian University, 30-060 Krakow, Poland; 5 Krakow Centre for Medical Research and Technologies, The St. John Paul II Hospital, 31-202 Krakow, Poland; 6 Department of Thromboembolic Disorders, Institute of Cardiology, Faculty of Medicine and Health Sciences, Medical College, Jagiellonian University, 30-060 Krakow, Poland

**Keywords:** perfluorooctanesulfonic acid (PFOS), thrombin generation, fibrin clot properties, environmental pollution, cardiovascular disease

## Abstract

Perfluorooctanesulfonic acid (PFOS) is a persistent organic pollutant linked in epidemiological studies to increased coronary artery disease (CAD) risk, higher LDL-cholesterol, hypertension, and other adverse outcomes. However, the mechanisms by which PFOS affects cardiovascular physiology, particularly coagulation, remain insufficiently understood. We evaluated the ex vivo effects of PFOS on fibrin clot structure and thrombin generation in platelet-poor plasma (PPP) and citrated whole blood from ten healthy volunteers (five women, aged 27–32 years; mean serum PFOS: 2.63 ± 0.85 μg/L). PPP samples were incubated with PFOS at 50, 200, and 400 μg/L. Assays included calibrated automated thrombogram, clot permeability (Ks), clot lysis time (CLT), thromboelastography (400 μg/L), and scanning electron microscopy (SEM). PFOS did not significantly modify endogenous thrombin potential or peak thrombin. In contrast, it reduced Ks and prolonged CLT at 200 and 400 μg/L, indicating impaired fibrinolysis. SEM images confirmed the formation of thinner, tightly packed fibrin fibers with reduced pore size at higher PFOS concentrations. These findings were consistent across dilution models, with only minimal changes observed in low-dilution protocols. Overall, PFOS appears to disrupt fibrin polymerization, generating denser and more fibrinolysis-resistant clots without major effects on thrombin generation. Such alterations may promote a prothrombotic state and predispose exposed individuals to clinically relevant thrombotic events, including myocardial infarction and stroke. Further studies are required to define the clinical consequences of PFOS-related coagulation abnormalities in exposed populations.

## 1. Introduction

Perfluorooctanesulfonic acid (PFOS) is a representative compound within the broad class of more than 4000 per- and polyfluoroalkyl substances (PFASs). These chemicals are characterized by exceptional thermal and chemical stability, resistance to metabolic degradation, and long environmental half-lives, leading to their classification as persistent organic pollutants capable of long-term bioaccumulation in human tissues [1,2,3,4,5]. PFASs have been extensively incorporated into consumer and industrial products, including food packaging, non-stick cookware, textile and paper coatings, water-repellent fabrics, personal care products, surfactants, and various manufacturing processes [1,2,3,4]. As a consequence, PFOS exposure is nearly ubiquitous, occurring primarily through ingestion, inhalation, and dermal absorption [4].

Large human biomonitoring studies conducted in Europe (HBM4EU) and the United States indicate that mean PFOS serum concentrations in the general population typically range from approximately 2.1 to 14.6 µg/L [5]. However, the literature also describes specific populations with substantially higher PFOS burdens, including residents of contaminated areas, occupationally exposed individuals, and populations with high consumption of fish and seafood, in whom serum PFOS concentrations may reach values in the hundreds of µg/L [2].

Although PFASs were once considered biologically inert, they have become the focus of intensive toxicological and epidemiological investigation. Growing evidence indicates that PFOS can disrupt systemic and cellular homeostasis, with reported associations including carcinogenesis, endocrine and metabolic dysfunction, immunomodulation, neurotoxicity, and other adverse health outcomes [1,2,3,4,5,6,7,8,9,10,11,12,13,14,15]. Of particular concern is the accumulating body of evidence linking PFOS exposure to cardiovascular pathology. Current epidemiological data indicate that elevated circulating PFAS concentrations are increasingly recognized as an emerging cardiovascular risk factor [1,2,3,4,5,6,7,8,9,10,11,12,13,14,15,16].

Several large epidemiological studies have reported associations between PFAS exposure and adverse cardiovascular outcomes, including dyslipidemia, hypertension, coronary artery disease, and incident cardiovascular events. Analyses from the NHANES cohort and the C8 Health Project have demonstrated significant associations between serum PFAS concentrations, elevated cholesterol levels, and increased prevalence of cardiovascular disease. Furthermore, prospective cohort studies conducted in Europe and the United States have linked higher PFAS exposure to an increased risk of hypertension and cardiovascular mortality [17,18,19,20,21,22,23,24,25,26,27,28,29,30,31,32,33,34,35,36,37,38]. A recent meta-analysis of 22 studies demonstrated statistically significant associations between PFAS body burden and the incidence of type 2 diabetes in cohort studies, although findings from case–control and cross-sectional studies were less consistent [37].

Despite these observations, the mechanistic pathways by which PFOS contributes to cardiovascular disease remain insufficiently defined. In particular, PFOS-induced alterations in haemostatic balance—especially perturbations of fibrin clot structure and fibrinolysis—represent a plausible but underexplored link between PFOS exposure and thrombotic cardiovascular events.

To address these knowledge gaps, this study aimed to determine whether PFOS directly alters fibrin clot architecture and thrombin generation in human plasma, thereby providing mechanistic insight into its potential prothrombotic effects.

## 2. Methods

### 2.1. Study Population

The study was conducted in accordance with the Declaration of Helsinki, and approved by the Jagiellonian University Bioethics Committee (1072.6120.154.2023 for EK—17 January 2024 and 1072.6120.79.2024 for KKr—11 September 2024).

Ten healthy adult volunteers (five women, aged 27–32 years), all affiliated with the medical staff of the institution. Informed consent was obtained from all subjects involved in the study.

Written informed consent has been obtained from the patients to publish the paper. All personal data of the study participant have been anonymized in a manner that ensures the individual can no longer be identified, directly or indirectly, in line with GDPR provisions.

Exclusion criteria included:

intentional exposure to elevated PFOS levels; current or recent infection (within the preceding 14 days) and/or a C-reactive protein (CRP) concentration ≥ 5 mg/L; a history of diabetes mellitus, pregnancy, active malignancy, or autoimmune disease; and the use of antithrombotic or anticoagulant medications, statins, or hormone therapies involving steroids or oestrogens.

### 2.2. Laboratory Measurements

Prior to final inclusion, the following laboratory tests were performed:

- serum PFOS concentration, measured using liquid chromatography–tandem mass spectrometry (LC-MS/MS), to exclude elevated levels due to unintentional exposure.
- CRP concentration, determined by immunonephelometry.
- oral glucose tolerance test (OGTT), to exclude diabetes and prediabetic conditions.

Following qualification, a single 20 mL sample of venous blood was collected from each participant into citrate tubes. Platelet-poor plasma (PPP) was prepared by centrifugation and used for subsequent analyses. Selected coagulation parameters were assessed in plasma samples both in the absence of PFOS (CAS No. 754-91-6) and following incubation with PFOS [HPC Standards GmbH, Borsdorf, Germany]. PFOS was dissolved in physiological saline and tested at three concentrations: 50, 200, and 400 µg/L. Accordingly, 50 µg/L represents the lower range of high or intermediate exposure, 200 µg/L reflects PFOS accumulation under chronic exposure conditions, and 400 µg/L allows assessment of biological effects at a high body burden, enabling evaluation of response characteristics (linear versus threshold-dependent) and the magnitude of changes in fibrin clot structure and function.

The following endpoints were evaluated:

- thrombin generation: endogenous thrombin potential (ETP), peak thrombin concentration (Peak) [nM], and lag time [min].
- fibrin clot properties: clot permeability (Ks) [×10^−9^ cm^2^] and clot lysis time (CLT) [min].

Thrombin generation was measured using the calibrated automated thrombogram (CAT) method. The protocol involved incubation of 500 µL PPP with 100 µL PFOS solution (final PFOS concentration = 400 µg/L), compared to control samples incubated with physiological saline. The same protocol was applied to assess clot permeability and lysis time at PFOS concentrations of 50, 200, and 400 µg/L.

Additionally, a second model was employed using lower plasma dilution (100 µL PPP + 5 µL PFOS/saline), while maintaining the same final PFOS concentrations, to confirm the reproducibility of results under minimal dilution conditions.

Given the possibility that PFOS may not directly affect plasma coagulation, thromboelastographic analysis was performed using citrated whole blood incubated with PFOS (400 µg/L) for 30 min at 37 °C, compared to control samples incubated with saline. This model (100 µL whole blood + 5 µL PFOS/saline) was designed to preserve minimal dilution and assess the contribution of platelet components to clot formation.

All assays were conducted in two independent replicates.

### 2.3. Scanning Electron Microscopy

To test the impact of PFOS on the structure of the fibrin clot, we used scanning electron microscopy. Clots were fixed using 2.5% glutaraldehyde, washed with distilled water, dehydrated in graded water–ethanol solutions, dried by the critical point procedure and sputter coated with gold. Samples were scanned in 10 different areas. EM images were taken with a ThermoFisher Scientific (Singapore) Helios 5 Hydra CX PFIB microscope. Images were obtained with landing energy adjusted to 500 V, 50 pA current, TLD detector (ThermoFisher Scientific) working in BD mode and immersion optics active.

### 2.4. Statistics

All statistical analyses were performed using appropriate methods to assess data distribution and compare group differences. Variables with normal distribution, as determined by the Shapiro–Wilk test, are presented as mean ± standard deviation (SD). Non-normally distributed variables are reported as median with interquartile range (IQR).

For pairwise comparisons, a paired sample *t*-test was applied when the assumption of normality was met. In cases where normality was not satisfied, the Wilcoxon signed-rank test was used as a non-parametric alternative for paired samples.

To evaluate differences across multiple PFOS concentration groups, one-way analysis of variance (ANOVA) was conducted. A *p*-value < 0.05 was considered statistically significant for all tests.

All statistical analyses were performed using GraphPad Prism version 10.4.0 (GraphPad Software, San Diego, CA, USA; licensed for AS).

## 3. Results

**Figure 1:**
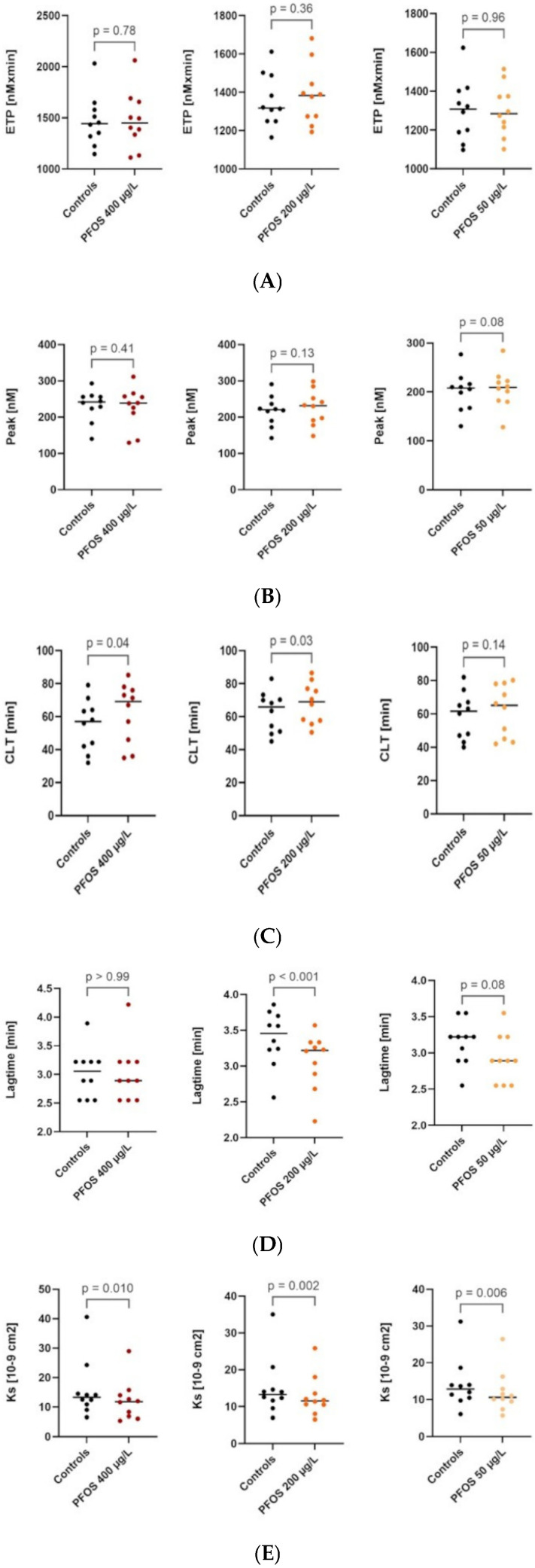
(**A**) Graphical presentation of plasma thrombin potential (ETP) in all PFOS concentrations compared to the control. (**B**) Graphical presentation of maximum generated thrombin concentration (Peak) in all PFOS concentrations compared to the control. (**C**) Graphical presentation of clot lysis time (CLT) in all PFOS concentrations compared to the control. (**D**) Graphical presentation of lag time for thrombin generation in all PFOS concentrations compared to the control. (**E**) Graphical presentation of clot permeability (Ks) generation in all PFOS concentrations compared to the control.

The mean serum PFOS concentration in the study population, measured before inclusion to exclude elevated levels due to unintentional exposure, was 2.63 ± 0.85 µg/L. In our model, no differences were observed in the parameters of thrombin generation using the CAT method, plasma thrombin potential (ETP), or maximum generated thrombin concentration (Peak) compared to the control [Table 1 and Figure 1A,B].

A significant reduction in clot permeability was observed at PFOS concentrations of 400, 200, and 50 µg/L compared with the control [Table 1; Figure 1A–E], whereas a marked prolongation of clot lysis time was evident at PFOS concentrations of 400 and 200 µg/L.

No differences were observed in endogenous thrombin potential or peak thrombin generation (Figure 1A,B); however, the lag time for thrombin generation was significantly shorter at PFOS concentrations of 200 µg/L compared to the control.

In the experiment, which was conducted on pooled frozen platelet-poor plasma using lower plasma dilution, no significant differences in thrombin generation were observed between control plasma and plasma containing 400, 200 and 50 µg/L PFOS, respectively (maximum deviations in individual measurements <6%). However, the average level of thrombin generation was lower than in samples diluted at a ratio of 500 µL platelet-poor plasma + 100 µL PFOS/saline (100 µL plasma + 20 µL PFOS/saline) by nearly 160 nM × min, indicating that this model is more suitable for evaluating the impact of tested substances in vitro.

In the lower plasma dilution model (100 µL plasma + 5 µL PFOS/saline), no effect of PFOS (at any concentrations used) on CLT was observed, and a slight reduction in Ks (up to −6.5%) in the presence of PFOS was noted without a clear trend relative to the concentration.

**Figure 2:**
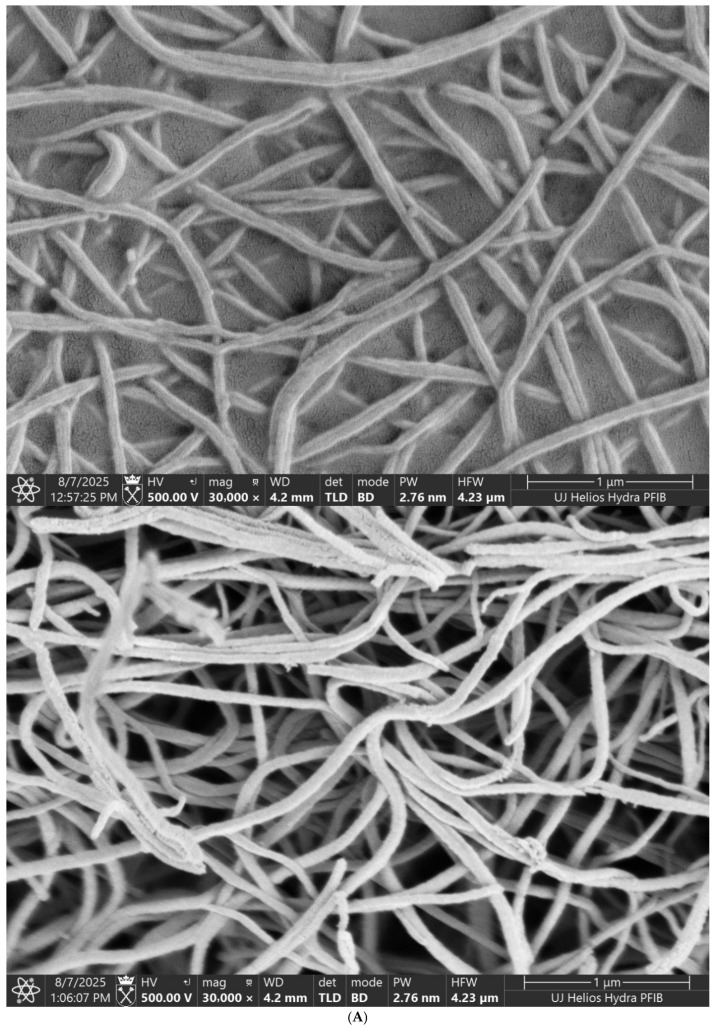

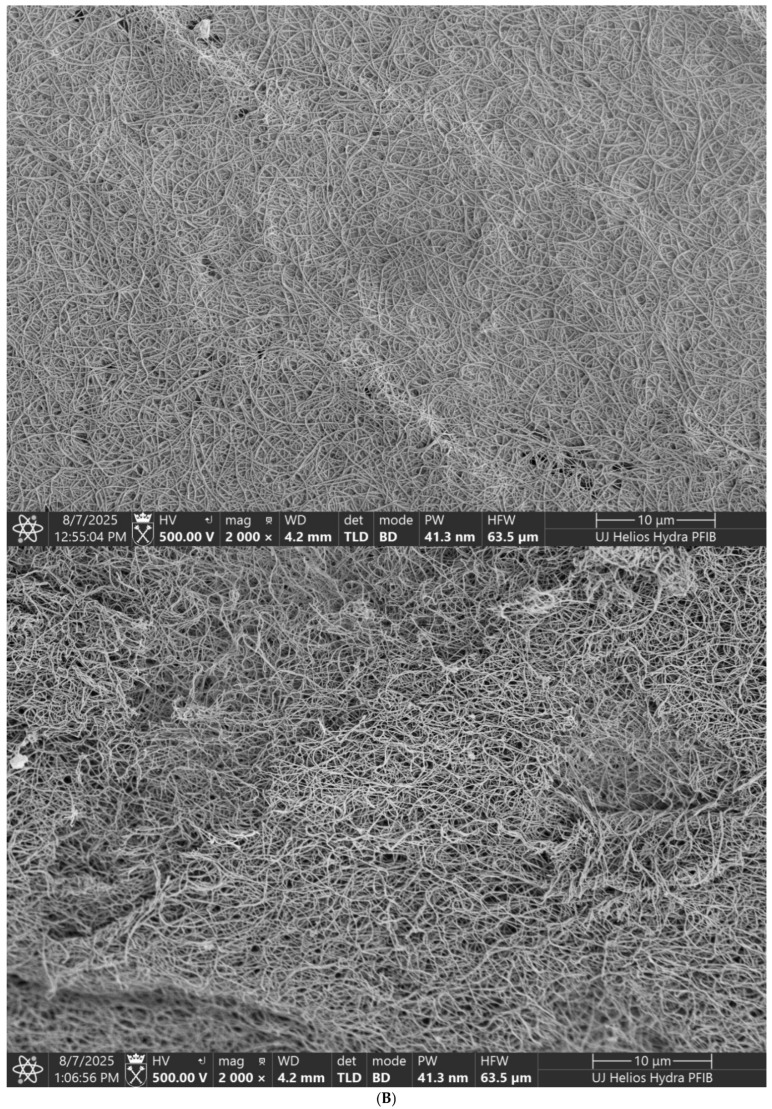

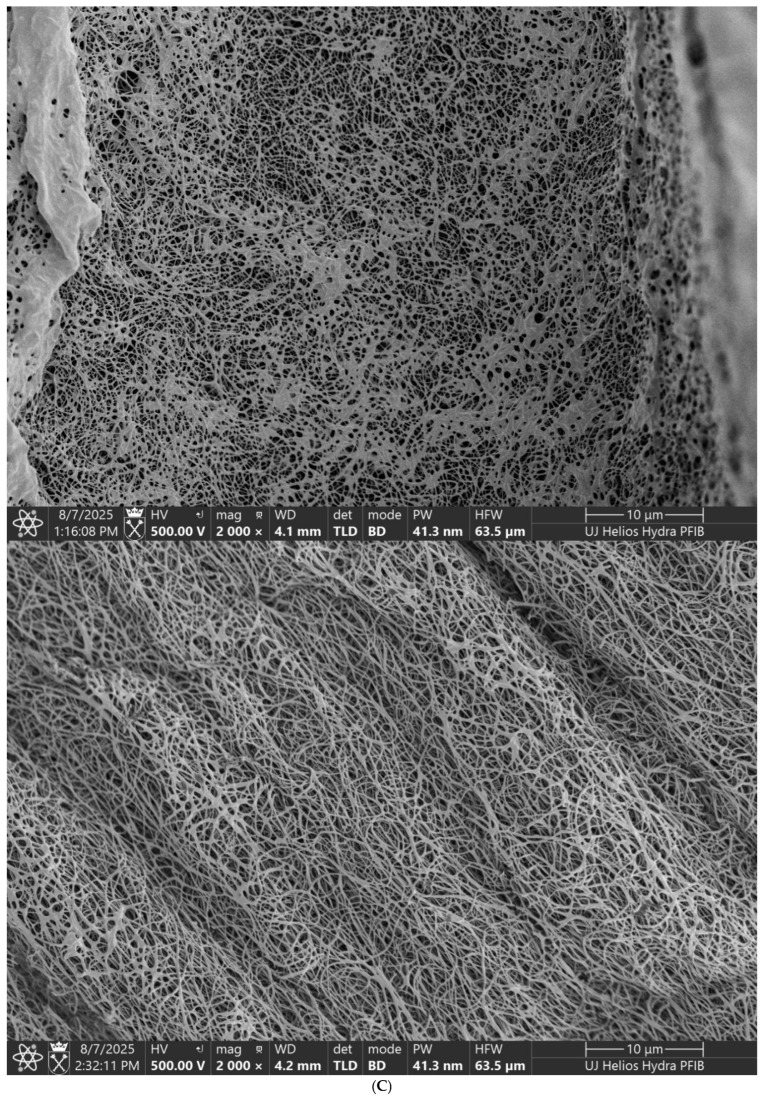
(**A**) structure of the fibrin clot areas at magnification of 30,000× for 400 ug/L PFOS concentrations compared to the control. (**B**) structure of the fibrin clot areas at magnification of 2000× for 400 ug/L PFOS concentrations compared to the control. (**C**) structure of the fibrin clot at magnification of 2000× for 200 ug/L PFOS concentrations compared to the control.

Scanning electron microscopy showed that fibrin clots exposed to higher PFOS concentrations (200 and 400 µg/L) had significantly altered ultrastructure, including reduced fiber diameter, increased density, and smaller pore size [Figure 2A–C)].

## 4. Discussion

Although traditional cardiovascular risk factors remain fundamental to clinical practice, they do not fully explain the residual risk observed in many patients. Increasing evidence highlights the importance of emerging determinants, including chronic inflammation, psychosocial and circadian stress, metabolic and immune dysregulation, and environmental exposures. Among these, environmental factors have gained particular clinical relevance. Exposure to environmental pollution—including ambient air pollutants, contamination of food and water, chronic noise exposure, extreme temperatures, and ionising or non-ionising radiation—is consistently associated with endothelial dysfunction, systemic inflammation, autonomic imbalance, and prothrombotic states. These pathophysiological mechanisms translate into an increased risk of hypertension, atherosclerosis, myocardial infarction, and stroke, underscoring the need to integrate environmental exposures into contemporary cardiovascular risk assessment and preventive strategies [24,25,26,27].

Among the various pollutants of concern, per- and polyfluoroalkyl substances (PFAS) have emerged as a class of persistent chemicals with potential cardiovascular effects.

The association between environmental exposure to PFAS and cardiovascular pathology remains an active area of investigation. To date, epidemiological studies have more consistently linked PFAS exposure with alterations in cardiovascular risk factors than clarified their direct biological effects [16,17,18,19,20,21,22,23,24,25,26,27,28,29,30,31,32,33,34,35,36,37,38,39].

As mentioned above, numerous studies conducted in adult populations have consistently reported that higher serum concentrations of PFOS and other PFAS are positively associated with the prevalence of atherosclerosis and traditional cardiovascular risk factors. However, these studies also indicate a lack of universally defined exposure thresholds for PFOS. Metabolic and cardiovascular complications are most commonly observed at serum concentrations exceeding approximately 5–10 ng/mL [18,19,20,21,22,23,24,25,26,27,28,29,30,31,32,33,34,35,36,37,38,39], although considerable interindividual variability in susceptibility exists, and adverse effects may occur at lower concentrations under conditions of chronic exposure in susceptible individuals.

Despite numerous observational analyses across diverse cohorts, the biological pathways underlying PFAS-related cardiovascular effects remain incompletely understood. Proposed mechanisms include increased systemic inflammation and oxidative stress [5,9,10,28,29,30,31,32,33,34,35,36,37,38,39].

Only a limited number of studies have examined the effects of PFAS on platelet activation or aggregation, and the available investigations vary substantially in analytical procedures and sample-handling methods, which hampers the comparability of their findings [40,41,42].

The present study was intentionally designed as a controlled in vitro investigation. The primary objective was not to estimate clinical risk but to mechanistically assess whether PFOS affects fibrin architecture and function under strictly standardised experimental conditions. Accordingly, considerations regarding sample size were guided by methodological requirements specific to in vitro toxicological research rather than by criteria applicable to clinical or epidemiological studies. Within this framework, the present study—representing, to our knowledge, the first direct demonstration of such effects—showed that exposure of human plasma to PFOS leads to a dose-dependent reduction in fibrin clot permeability, accompanied by a significant prolongation of clot lysis time.

The PFOS concentrations selected in this study were based on established principles of experimental toxicology, in which a range of concentrations—including values exceeding the population mean—is commonly applied to reveal potential biological effects, assess dose—response relationships, and avoid false-negative findings at low exposure levels. Importantly, all concentrations used in the present study have been reported in the serum of exposed individuals in the general population [34,36]. Notably, the observed alterations in fibrin clot structure and function were predominantly induced at higher PFOS concentrations (200–400 µg/L).

In addition, PFOS exerted a more pronounced effect on the structural rather than the functional properties of fibrin clots. Scanning electron microscopy revealed that clots formed in the presence of PFOS consisted of thinner, more densely packed fibres with increased branching. These structural abnormalities were most evident at higher PFOS concentrations and resulted in markedly reduced clot permeability and increased resistance to fibrinolysis.

Importantly, thrombin generation was not significantly altered by PFOS, supporting the hypothesis that the observed effects arise from direct physical interactions between PFOS and polymerising fibrinogen rather than from modulation of the tissue factor–dependent coagulation pathway [43,44,45,46,47,48]. The proposed mechanism remains hypothetical and is based on indirect observations, including the well-documented affinity of PFOS for plasma proteins and the known sensitivity of fibrin polymerisation to subtle physicochemical perturbations.

Importantly, even weak non-covalent interactions may substantially influence fibrin architecture and subsequent fibrinolysis; thus, the observed effects are more likely mediated through PFOS-induced alterations in fibrin structure rather than through direct interference with fibrinolytic enzymes. Confirmation of direct PFOS–fibrinogen interactions would require dedicated biophysical studies, which were beyond the scope of the present work.

This interpretation is further supported by the use of thrombin as the coagulation activator in the permeability assay, which effectively isolates fibrinogen–PFOS interactions from upstream coagulation processes.

The alterations in fibrin architecture identified in this study are well-recognised markers of a prothrombotic tendency and have been linked to an increased risk of thrombotic complications in patients with pre-existing atherosclerotic disease [49]. Several prospective studies have shown that a fibrin clot phenotype characterised by a dense fibrin network is associated with an increased risk of cardiovascular events in both short- and long-term follow-up, independent of traditional cardiovascular risk factors.

For example, in the prospective PLATO study of 4354 patients with acute coronary syndromes, impaired fibrin clot structure and function were associated with a 20% increase in the relative risk of cardiovascular mortality (HR 1.20, 95% CI 1.01–1.40) and a 21% increase in all-cause mortality (HR 1.21, 95% CI 1.03–1.42) [50]. Other large cohort studies have demonstrated that this prothrombotic fibrin clot phenotype is an independent risk factor for recurrent venous thromboembolism and pulmonary embolism [51,52,53]. One such cohort study of 320 patients aged 18–70 years after a first episode of venous thromboembolism showed a recurrence rate of 25%, with 12% of patients exhibiting reduced clot permeability and a shortened lag phase, indicating accelerated clot formation [51].

Furthermore, clinical studies have shown that, in patients with acute ischaemic stroke, the predisposition to form dense fibrin clots is significantly correlated with the severity of neurological deficits [54].

Taken together, these findings suggest that changes in fibrin clot architecture and function may have clinically important implications. Nevertheless, extrapolation of the in vitro effects of PFOS observed in this study to clinical outcomes should be undertaken with caution and requires confirmation in well-designed in vivo and clinical investigations.

It is also worth noting that experimental studies have demonstrated that certain commonly used medications, such as statins and aspirin, as well as genetic factors, inflammatory states, oxidative stress, and coexisting comorbidities, can modulate fibrin clot function and phenotype. Importantly, these structural and functional modifications have been associated, in clinical studies, with a reduction in mortality among patients with thromboembolic complications [55,56,57,58,59,60,61,62].

Several limitations merit consideration. As an ex vivo experimental study, the design cannot fully replicate the complex physiological environment of the human circulation. Laboratory methods, although performed using rigorous protocols and duplicated measurements, are susceptible to random variability. Moreover, PFAS exposure in real-world settings is typically chronic and occurs alongside multiple co-exposures and comorbidities, which may modify biological responses in ways not reproducible in controlled experimental systems. Finally, numerous unmeasured factors may contribute to inter-individual susceptibility.

To mitigate confounding, the study included only healthy young volunteers without comorbidities or medication use and with no known recent exposure to elevated PFOS levels. Baseline PFOS concentrations were measured in all participants to ensure accurate characterization of exposure. Nevertheless, the possibility of residual variability cannot be excluded.

## 5. Conclusions

Our study demonstrates that PFOS directly alters fibrin clot architecture, producing a denser and less permeable fibrin network with impaired susceptibility to fibrinolysis. These structural modifications are typical of a prothrombotic phenotype and may increase the risk of thrombotic events, especially in individuals with cardiovascular or systemic prothrombotic conditions, such as acute coronary syndromes, ischemic stroke, pulmonary embolism, or peripheral artery disease. These findings provide mechanistic support for epidemiological observations linking PFAS exposure with adverse cardiovascular outcomes, and highlight the need to investigate whether chronic, real-world PFAS exposure contributes meaningfully to thrombotic risk in vulnerable patient populations such as those with established cardiovascular disease, diabetes, cancer, or chronic inflammation. Future research should expand this work to other PFAS compounds and to broader components of the coagulation and fibrinolytic systems.

Given the persistence of PFAS in the environment, complete elimination of exposure is unlikely. Therefore, systematic monitoring, regulatory reduction of contamination sources, and continued public health strategies aimed at minimizing individual PFAS burden remain essential to mitigate potential cardiovascular consequences.

## Figures and Tables

**Table 1:** Group comparison between controls and all PFOS concentrations.

Variable	Controls *	PFOS 400 µg/L	*p* Value *	PFOS 200 µg/L	*p* Value **	PFOS 50 µg/L	*p* Value ***
**Ks [10^−9^ cm^2^]**	15.1 ± 8.02	12.2 ± 6.88	**0.001**	12.8 ± 5.52	**0.002**	12.1 ± 5.81	**0.006**
**CLT [min]**	58.8 ± 14.0	62.5 ± 18.0	**0.04**	68.2 ± 12.3	**0.03**	62.0 ± 15.4	0.14
**Lag time [min]**	3.22 (2.89–3.55)	2.89 (2.55–3.22)	**0.99**	3.22 (2.84–3.33)	**<0.001**	2.89 (2.55–3.22)	0.08
**ETP [nMxmin]**	1330 (1242–1492)	1449 (1285–1664)	0.78	1383 (1261–1481)	0.36	1285 (1200–1399)	0.96
**Peak [nM]**	217 ± 42.4	227 ± 56.3	0.41	226 ± 47.2	0.13	207 ± 40.2	0.08
**ttPeak [min]**	6.72 ± 0.88	6.5 ± 0.9	0.51	6.72 ± 0.75	**0.008**	6.38 ± 0.70	0.07

Control values were taken from each set of experiments (10 controls vs. 10 PFOS). Endogenous thrombin potential (ETP), peak thrombin concentration (Peak) [nM], lag time for thrombin generation (Lag time) [min], and properties of the fibrin clot—clot permeability (Ks) [×10^−9^ cm^2^] and clot lysis time (CLT) [min]. * Represents a difference between controls and PFOS 400 ug/L. ** Represents a difference between controls and PFOS 200 ug/L. *** Represents a difference between controls and PFOS 50 ug/L.

## Data Availability

The original contributions presented in this study are included in the article. Further inquiries can be directed to the corresponding author.
